# Are We on the Right Track for HCV Micro-Elimination? HCV Management Practices in Dialysis Centers in Poland—A National Cross-Sectional Survey

**DOI:** 10.3390/jcm12072711

**Published:** 2023-04-04

**Authors:** Paulina Czarnecka, Kinga Czarnecka, Olga Tronina, Teresa Baczkowska, Weronika Zarychta-Wisniewska, Magdalena Durlik

**Affiliations:** 1 Department of Transplantation Medicine, Nephrology and Internal Diseases, Medical University of Warsaw, 02-006 Warsaw, Poland; 2 Department of Immunology, Transplant Medicine and Internal Diseases, Medical University of Warsaw, 02-006 Warsaw, Poland

**Keywords:** hepatitis C virus (HCV), eradication of infection, Direct Acting Antivirals (DAA)

## Abstract

Chronic hepatitis C (CHC) is prevalent in the hemodialysis-dependent population. Currently, all patients with CHC should be considered for treatment; however, many hemodialysis-dependent patients are still left untreated. Following HCV cure, accurate surveillance is mandatory to reduce liver-related mortality and prevent reinfection. We aimed to establish HCV management practices and barriers to HCV elimination in dialysis centers in Poland. Polish dialysis centers were surveyed via email. The HCV management strategies were investigated. Representatives of 112 dialysis centers responded, representing 43.1% of all dialysis centers in Poland and 43.4% of hemodialysis-dependent patients’ volume. Most respondents were Heads of hemodialysis centers and board-certified nephrologists. The study demonstrated that in the vast majority of hemodialysis centers (91.6%), subjects are considered for antiviral treatment (AVT); however, many obstacles preventing patients from being prescribed AVT were identified; patients’ reluctance to undergo AVT was most reported (60%). The majority of dialysis units neither evaluate patients with CHC for liver fibrosis (60.4%) nor screen them for hepatocellular carcinoma (53.5%). In conclusion, the presented study demonstrates that HCV management practices across Polish dialysis centers vary substantially. There is a need to optimize and streamline the HCV management infrastructure in the hemodialysis population in Poland.

## 1. Introduction

Chronic hepatitis C (CHC) is a major health concern globally, affecting 71 million people worldwide and 140,000 patients in Poland, significantly contributing to liver-related mortality [1,2,3,4]. Now that effective antiviral treatment (AVT) is available, a CHC eradication target was set by the World Health Organization (WHO): they proposed that along with a reduction of the rate of new infections, hepatitis C virus (HCV)-related mortality is to be reduced by 65% [5]. Viral eradication has been previously demonstrated with smallpox, which was made feasible through a mass vaccination campaign. Given the lack of an HCV vaccine, this goal may be pursued only with a comprehensive approach toward HCV elimination.

The WHO’s aim was to achieve macro-elimination via micro-elimination, which is less complex and more cost-effective. Micro-elimination aims to eradicate HCV by addressing special population groups that have a known higher HCV prevalence rather than merely screening the entire population; hemodialysis-dependent patients are one of the population groups targeted [6]. Despite the excellent efficacy of AVT, the current goal is hardly attainable.

CHC is far more prevalent in hemodialysis (HD)-dependent patients as they undergo frequent invasive medical procedures and are prone to nosocomial spread; consequently, the risk increases with time spent on dialysis [6]. The most recent report on the dialysis status in Poland demonstrated that the number of hemodialyzed patients exceeded 18,000 with a 3.8% HCV seroprevalence: 10-fold more than in the general population [7].

CHC is a well-recognized risk factor for increased liver-related morbidity and mortality in patients on renal replacement therapy (RRT), and successful HCV elimination improves clinical outcomes. Additionally, CHC in HD-dependent patients negatively affects overall survival and results in an increased risk of cirrhosis, HCC, and a lower quality of life compared to their HCV-negative counterparts [6,8,9]. Therefore, all patients with end-stage renal disease (ESRD) should be considered for AVT [1,10,11].

Former interferon-based therapies were neither effective nor well-tolerated, which deterred from AVT commencement [6]. With the advent of oral direct-acting antiviral agents (DAAs), which are highly effective and well-tolerated, the landscape has dramatically changed. Currently, a few treatment options are available for patients with CHC on RRT, including a fixed-dose combination of glecaprevir and pibrentasvir, sofosbuvir and velpatasvir or grazoprevir and elbasvir, not requiring dose adjustments [1,10]. The latter option has applications only for genotype 1b of HCV. In cases of decompensated cirrhosis and severe renal impairment, a fixed-dose combination of sofosbuvir and velpatasvir, without ribavirin, for 24 weeks is recommended. Despite the advent of DAAs, patients on hemodialysis have been reportedly left untreated for reasons not yet elucidated [8].

As aforementioned, eradication of HCV requires a comprehensive strategy to succeed; therefore, efforts are made not only for HCV treatment but also for the prevention of HCV transmission. Despite the implementation of multiple precautions, HCV spread within dialysis centers continues, with the prevalence of HCV as a contributing factor [11,12,13,14]. The prevalence of HCV may be reduced with DAAs; however, the risk of reinfection persists if the patient remains on maintenance hemodialysis. However, little is known about the management practices of patients on hemodialysis, from a nephrological standpoint, after they achieve sustained virologic response (SVR) and whether these practices prevent HCV reinfection.

Disease progression of CHC results in liver inflammation and fibrosis, which may be mitigated to a certain extent by DAAs [15]. It should be highlighted that HCV eradication constitutes only the first step because not all subjects who eradicated the virus are entirely cured; long-accumulated histopathological changes may persist. Therefore, continued surveillance of cured patients is mandatory to prevent undermining efforts put forth for HCV eradication. This can be achieved through the evaluation of fibrosis. It has been documented that liver fibrosis can be utilized for the prognostication of adverse outcomes, patient mortality and as a predictor of decompensation or hepatocellular carcinoma (HCC) occurrence [16,17]. It has also been reflected in the most recent Kidney Disease: Improving Global Outcomes (KDIGO) Work Group guidelines which recommend that all patients with chronic kidney disease (CKD) and CHC be evaluated for fibrosis using noninvasive biomarkers, such as fibrosis-4 (FIB-4), aspartate transaminase-to-platelet ratio index (APRI), or transient elastography [11].

CHC is a major cause of the occurrence of HCC [18]. Patients with CHC and those that achieved SVR but have advanced fibrosis or cirrhosis should undergo regular screening for HCC with ultrasound and alpha-fetoprotein (AFP) assay [1,10]. Knowledge of the fibrosis stage enables better HCC stratification and optimization of HCC surveillance.

The insufficient donor supply available for transplantation, the growing needs in this matter, and access to effective AVT have encouraged transplant centers to broaden donor pools by utilizing HCV viremic donors. There is no agreement across transplant centers on whether this direction is right; however, it has been supported by the KDIGO guidelines [11]. Considering HCV elimination efforts, this additional pool of patients will become limited with time; hence, it is crucial to use this time wisely for the benefit of HD-dependent patients.

The aim of this study was to investigate the HCV management practices across dialysis centers in Poland and identify potential barriers that prevent us from reaching the goal of HCV elimination by 2030. We strongly believe that identifying obstacles could be the first step toward HCV elimination in the ESRD population in the authors’ country.

## 2. Materials and Methods

### 2.1. Study Administration

The study was conducted between January and December 2022. Both private and public adult hemodialysis (HD) centers in Poland, which were active in 2022 (*n* = 260), were approached for the survey via email, and only one representative (medical doctor) of each unit was to complete the survey. Each HD center was represented only once. Questionnaires were administered to the head of the HD unit. Only they had the authority to complete the form unless it was specifically requested by the HD center to assign another email address to them. In this case, the access of the previous email address owner to the questionnaire was revoked. The survey was performed anonymously, and the questionnaire was blocked once the form was received from the respective addressee to prevent double submissions. The HD centers were notified of the survey thrice. The questionnaire consisted of 14 multi-choice, semi-open questions.

The study was conducted in line with the tenets of the Declaration of Helsinki and was approved by the Ethics Committee of the Medical University of Warsaw (AKBE/205/2021).

### 2.2. Statistical Analysis

The data were analyzed using IBM SPSS Statistics version 25. A series of frequency analyses and χ^2^ or exact Fisher Tests were performed. *p* values of < 0.05 were considered significant. An χ^2^ or exact Fisher test was used to study differences between public and private facilities and between tertiary and secondary units. The effect size was measured with the V Cramer coefficient.

The results are presented as percentages and frequencies or means and standard deviations, whichever was appropriate. Percentages were calculated using the overall number of valid responses to each question as the denominator. If the respondent left the question blank, it was excluded from the denominator. For multiple-choice questions, the number of participants responding to that particular question constituted the total number in the denominator. Consequently, for these questions, the column totals exceeded 100%.

## 3. Results

A total of 112 HD centers responded, representing 43.1% of all HD centers in Poland. The majority of them were private (*n* = 79, 70.5%), with an overall 8080 (28–176 pts) patients being managed within the surveyed facilities; this patient count accounts for 43.4% of the patient volume that is on maintenance hemodialysis in Poland. Out of 33 public facilities, 11.6% constituted tertiary units. The surveyed professionals were mainly heads of the HD centers and board-certified nephrologists (Table 1).

### 3.1. Detailed Survey Questions

#### 3.1.1. Do You Refer Patients with CHC for AVT as a Routine Practice?

Five responders, all from public facilities, did not provide an answer to the question. Of the remaining facilities, 91.6% (*n* = 98) claimed to refer HD patients for AVT as a routine practice, whereas 8.4% (*n* = 9) reported otherwise (Table 2).

#### 3.1.2. Where Are Subjects Typically Referred to for AVT?

The vast majority of respondents (83.9%, *n* = 94) refer CHC individuals to infectious disease outpatient clinics to be evaluated for AVT, followed by hepatology outpatient clinics (17%, *n* = 19) (Table 2).

#### 3.1.3. Why Are Hemodialysis Patients with CHC Not Considered for AVT?

Eleven HD centers did not answer the question and declared not to have had CHC patients recently and were not able to answer the question based on their current population experience. Within the remaining number of HD centers, twenty-eight units (27.7%) declared to consider all subjects for AVT. The primary reason for not referring patients on RRT for AVT was patients’ unwillingness to undergo CHC treatment (*n*= 41, 40.6%), followed by contraindications to AVT (*n* = 19, 18.8%) and short life expectancy (*n* = 11, 10.9%). Lack of knowledge of CHC management and potential AVT-induced adverse reactions was reported as a deterrent factor to AVT in 8.9% (*n* = 9) and 6.9% (*n* = 7) of cases, respectively. Organizational matters, the most reported being the distance to the outpatient clinic and lack of availability of DAAs, were each reported in 5.9% (*n* = 6) of cases (Table 2).

#### 3.1.4. Do You Evaluate Patients with CHC for Liver Fibrosis within the HD Center?

Lack of routine liver fibrosis assessment in patients with CHC prevailed (60.4%). Forty dialysis units declared to assess liver fibrosis; however, this was limited to 75% in subjects referred for AVT only.

#### 3.1.5. Do You Screen Patients with CHC for HCC within the HD Center? What Is Your HCC Surveillance Model for Patients with CHC?

HCC surveillance was exercised in as many as 47 dialysis centers (46.5%), while 54 (53.5%) confirmed not having HCC surveillance for patients with CHC. The predominant HCC surveillance model was an ultrasound examination every six months followed by AFP with the same frequency (Table 2).

#### 3.1.6. Where Are Dialyzed Patients following Successful HCV Eradication?

Seven HD centers did not provide an answer to the question. Virtually half of the respondents (46.7%, *n* = 49) dialyzed their patients—after they achieved SVR—along with HCV-naïve individuals, whereas the second most common practice (40.9%, *n* = 43) was the utilization of dedicated machines for HCV-viremic patients (Table 2).

#### 3.1.7. Would You Offer an HCV Viremic Kidney to an Aviremic Recipient?

Fifty-four responders (48.2%) declared to consider offering HCV-viremic kidney allografts to HCV-aviremic kidney transplant candidates, and the remaining centers did not consider such an option. Fear of potential complications following kidney transplantation (KTx) from an HCV-viremic donor to an HCV-negative recipient (HCV NAT D+/R− was a prominent deterrent factor (*n* = 39, 72,2%), followed by lack of confidence in the efficacy of DAAs after kidney transplant (*n* = 27, 50%) (Table 2).

### 3.2. Differences in HCV Management Practices between Private and Public HD Centers

There were no significant differences between private and public dialysis centers in terms of patients with CHC being referred for AVT (*n* = 92.2% vs. 90.0%, respectively; *p* = 0.708). Similarly, no differences were found in reasons for not referring patients with CHC for AVT; however, compared to private centers, a lack of knowledge on AVT was more often reported from public centers, with the effect size being small (3.8% vs. 18.2%, respectively, *p* = 019, V = 0.24) (Table 3).

Routine liver fibrosis or HCC surveillance practices did not vary between the public and private dialysis centers (*p* = 0.656, χ^2^(1) = 0,20; *p* = 0.266, χ^2^(1) = 1,24, respectively). However, when detailed HCC surveillance protocols were analyzed, public centers declared that they performed USG examination and AFP every 6 months, which was significantly more often compared to private centers (USG, 33.3% vs. 16.5%, χ^2^(1) = 3.94, *p* = 0.047, *V* = 0.19; AFP, 24.2% vs. 6.3%, *p*= 0.019, V = 0.26, respectively) (Table 3).

When asked about the dialysis machine used for the management of patients with CHC after they achieved SVR, no significant differences were reported (χ^2^(2) = 1.96; *p* = 0.352) (Table 3).

### 3.3. Differences between Hemodialysis Centers within Tertiary and Secondary Hospitals in Poland

No statistical difference was noted in terms of HD centers referring patients with CHC for AVT within the tertiary and secondary hospitals (*p* = 0.279) (Table 4). Similar reasons were also found for not considering the patients on RRT for AVT (Table 4).

Significantly more tertiary institutions assessed liver fibrosis in patients with CHC than their secondary counterparts (*p* < 0.001, V = 0.74). Attitude towards the HCC survival protocol was similar in the tertiary and secondary hospitals (χ^2^(1) =1,11, *p* = 0.293), and so was the machine used for subjects who eradicated the virus *p* = 0.882 (Table 4).

## 4. Discussion

This national survey is the first to analyze HCV attitudes and care practices in Poland. This study identified noteworthy differences in HCV management strategies across HD centers in Poland, along with the vulnerability of the national HCV infrastructure in terms of the ESRD population.

Surveyed HD centers reported to have 316 anti-HCV positive patients, including 101 with active viremia, which constitutes 3.9% and 1.25%, respectively, of the total hemodialysis population volume in Poland. This is congruent with the most recent national report on hemodialysis status in Poland, in which the estimated prevalence of anti-HCV prevalence was 3.8% and active viremia 1.1%. This may underscore the fact that the sample was representative [7].

A vast majority of surveyed HD centers declared that they routinely refer patients for AVT, and only less than 10% did not. Nevertheless, of the HD centers referring patients for AVT, 46% pointed to obstacles hindering patients from actually being treated. The primary reason for the lack of AVT commencement was patients’ reluctance to undergo therapy, followed by organizational barriers and/or lack of DAAs availability. In such a scenario, an impressive percentage of HD centers that consider patients for AVT may seem to be overly optimistic and may not yield desirable effects in the form of HCV elimination. Currently recommended AVT schedules with reduced pills burden and short treatment duration are quite convenient from the patient’s perspective; therefore, we may speculate that the subject’s averseness, at least to some extent, stems from a lack of expertise among dialysis physicians or being hesitant towards AVT themselves which was documented in more than 20% of our responders and may result from previous interferon experience. This issue has been likewise raised in other papers [19,20]. Importantly, it has been previously documented that patients on maintenance hemodialysis mostly rely on their dialysis physician’s opinion on medical-related matters. A more granular analysis of patient reluctance is required to fully elucidate the root cause and address it with relevant corrective measures.

Surprisingly many responders pointed to contraindications to AVT as a reason for not treating the patient compared to drug-drug interactions. With the current AVT armamentarium, contraindications are very limited and mostly related to interactions with concomitant medication, e.g., anticonvulsants. We may presume that those results may similarly stem from little expertise on the current CHC treatment landscape. Owing to the anonymous nature of the survey, we were not able to verify with responders which contraindication they were referring to specifically.

Our survey indicates that most HD centers refer their patients to infectious disease outpatient clinics to be evaluated for AVT. Given the specificity of the ESRD population, distance to the outpatient clinic and long waiting time for the appointment may be a deterrent factor and bottleneck for HCV elimination, especially since the time to the first visit in an infectious disease clinic may exceed 9 months in some regions. Polish HD centers do not have direct access to DAAs; therefore, the only solution is to await specialist consultation, resulting in linkage to care being often unsuccessful.

Now that the general recommendation for AVT may be applied to HD-dependent subjects, the population should be an easy target for HCV eradication. Nonetheless, the current HCV care model needs to be simplified to ensure optimal coordination of treatment and to reduce waiting time. During their most recent congress, the WHO also urged towards a similar direction, pointing to decentralization and radical simplification of hepatitis care [21].

We strongly believe that linkage to care could be improved by engaging dialysis physicians in the DAA-prescribing process. Now that highly effective AVT with impressive tolerability is available, the trend towards decentralization and task-sharing approach in HCV management is justifiable, especially since HCV management may be successful regardless of the level of expertise of the treating physician [22]. Moreover, nephrologists taking the initiation may streamline care and reduce the number of visits outside the dialysis unit and referrals to specialists, shorten the time to AVT initiation, and ensure close monitoring of the treatment within the HD center.

Undoubtedly, not all AVT could be coordinated within the HD unit; however, there is a pool of patients, such as treatment-naïve patients or those without decompensated cirrhosis, who could be successfully treated within HD centers with great success. The most demanding patients, i.e., DAA non-responders or those with decompensated cirrhosis, could be referred to an infectious disease specialist/hepatologist. Detailed suggestions on possible solutions to HCV management practices improvement have been summarized in Table 5.

Many tools and solutions facilitate HCV management in hepatitis non-expert settings. First, the American Association for the Study of Liver Diseases and the Infectious Diseases Society of America (AASLD/IDSA) came forward with a simplified treatment guidance for HCV-naïve individuals and those with compensated cirrhosis, which with remote support from a hepatitis specialist was proved to be successful [10,23]. Such remote consultations could be arranged between hepatitis experts and dialysis physicians, which could be more efficient than the referral system. Furthermore, the use of pangenotypic agents does not mandate regiment adjustment per genotype, and no dose adjustment is needed in the ESRD population if the European Association for the Study of the Liver (EAS)L or AALSD/IDSA guidelines are followed [1,10]. Drug-drug interactions between DAAs and other medications may be successfully verified using the University of Liverpool online tool (hep-druginterations.org/checker). Liver stiffness may be assessed with routine blood tests, Fib-4, and APRI if transient elastography is not available [1,10].

Nonetheless, delegating AVT to HD centers requires a suitable infrastructure, including funds and training allocation. The amount of paperwork and time constraints may hamper nephrologists’ involvement.

More than half (60%) of the HD centers claimed that they did not assess liver fibrosis routinely in patients with CHC, while in another 30%, fibrosis was assessed only in patients prescribed AVT. Given the above and the multitude of barriers reported in our survey preventing HD-dependent patients from receiving AVT, many individuals may be left untreated without accurate liver fibrosis assessment, despite continued HCV-related abnormalities accumulation. Importantly, fibrosis may serve as a predictor of decompensation or of HCC in patients with CHC, which is why it is important to monitor liver stiffness as recommended by KDIGO [11,16,17].

Liver fibrosis assessments may be even more critical for HD-dependent patients waitlisted for a kidney transplant. It has been previously demonstrated that HD-dependent patients may have liver injury without aminotransferase elevation, as they do not reflect the liver injury decisively in this population. Advanced fibrosis does not exclude patients from receiving kidney transplants; however, it may pose a risk of portal hypertension-related complications. Therefore, fibrosis assessment in patients with known liver injury would facilitate the discussion and decision-making process in terms of AVT, particularly in patients who decline the opportunity for treatment. Another subset of patients who could benefit from liver fibrosis assessment would be kidney transplant candidates, if diagnosed with cirrhosis with indirect fibrosis indices prior to transplantation, who could qualify for both a liver-kidney transplant rather than a kidney transplant alone. Given the progressive nature of liver fibrosis in HCV-infected individuals, some authors have pointed out the need for repeated liver fibrosis assessments in HD-dependent patients waitlisted for KTx, with the assessments conducted at a frequency based on the initial score.

Moreover, responders pointed to a lack of equipment for liver fibrosis assessment, whereas recommendations clearly state that, prior to AVT, liver fibrosis assessment may be based on both transient elastography and routine biochemical results without any dedicated equipment [1,10]. Therefore, we may speculate that the lack of liver fibrosis assessment implies a lack of awareness of available tools and the importance of fibrosis assessments among dialysis physicians.

Furthermore, the majority of responders declared that their facilities do not routinely impose HCC surveillance protocols in patients with CHC, including six HD units that screen patients for HCC only when HBV/HCV coexist and another five that only screen patients with cirrhosis; two replied that they screen purely on infectious disease outpatient clinic recommendations without having an internal HCC surveillance protocol. Others pointed out that currently, they only have HCV-cured patients under their care, which may indicate that, in their view, this population does not require oncological surveillance.

Importantly, the EASL and AASLD recommend HCC screening in the CHC untreated population; however, all subjects following SVR with advanced fibrosis at baseline (F3,F4) should also be screened for HCC with ultrasound examination bi-annually with or without AFP [1,10].

Given the reluctance to undergo AVT, a substantial number of patients may be subjected to prolonged active viremia, resulting in continued liver damage. HCC diagnosis may be made at more advanced stages with a poor prognosis and limited treatment options when the abovementioned factors are coupled with a lack of mandatory fibrosis evaluation.

The WHO ’s goal of reducing HCV-related morbidity and mortality cannot be ascertained without proper fibrosis and HCC surveillance. There is a need to improve nephrologist awareness of HCV care standards to allow for knowledgeable patient management in this area.

Virtually half of the responders declared that they managed patients with CHC following SVR on machines that were dedicated for patients with hepatitis, while some placed them separately on dedicated machines for patients that achieved SVR. Interferon-based therapies with a high risk of viral relapse, as seen previously, justified such practices. However, with the availability of DAA, the approach does not stand to reason. Furthermore, isolation of HCV viremic patients has not been firmly confirmed as an effective measure to prevent HCV spread, but in certain circumstances, for example, low patient:personnel ratio, it may be justified [9,24,25]. Epidemiological investigations have shown that patients dialyzed nearby are at a greater risk of HCV infection than those dialyzed on the same machine as HCV viremic ones [9,14]. This may imply that non-adherence to mandatory precautions and not the machine itself is an obstacle to eradicating CHC; this factor requires due consideration rather than mere isolation of patients with CHC.

It should be noted that patients who achieve SVR with interferon-free regimens are free from the virus, with viral relapse being highly unlikely, which is reflected in CDC guidelines that recommend the management of such patients along with HCV-naïve counterparts [12]. The KDIGO advocates against the isolation of HCV-infected patients in HD settings [11]. Importantly, isolating HD-dependent patients that achieved SVR from HCV viremic individuals may be even more harmful. This not only creates an impression that they have not been cured of the virus and deters other viremic patients from AVT but also puts them at greater risk of reinfection, especially in case of faulty infection control precautions and regular HCV RNA testing not being a part of routine practice. Managing patients who eradicated the virus on dedicated machines is unjustifiable and may produce unnecessary organizational burdens.

Patients who have been successfully cured of the virus should be dialyzed along with HCV-naïve patients with universal precautions measures being respected, and this is most effective in preventing within-unit HCV spread, whereas separating patients with HCV is illegitimate.

Currently, the utilization of HCV viremic organs is increasing [26]. This is mainly driven by the opioid epidemic, a shrinking donor pool, and a long waitlist time. The present study shows that virtually half of the responders were comfortable with offering HCV viremic organs to potential organ recipients; however, the majority allowed such an option only in HCV viremic recipients. We agree that HCV viremic organs should be considered for HCV viremic recipients as the first preference; however, naïve recipients should not be deprived of this choice, especially in centers with long waitlist times or highly immunized kidney transplant candidates. This approach is congruent with KDIGO’s most recent guidelines, highlighting that all kidney transplant candidates should be considered for an HCV-infected allograft, regardless of their HCV status [11].

In the authors’ country, this scenario is currently unlikely because donors are not routinely verified for viremia with HCV RNA NAT assay prior to transplantation, and only patients with CHC can be offered anti-HCV + organs, thereby preventing the determination of the actual risk of transmission and informative decision-making. We may speculate that up to 40% of anti-HCV + donors could be aviremic and could donate an organ to aviremic recipients with a marginal risk of viral transmission, given the spontaneous HCV eradication rate.

Among opponents of HCV NAT D+/R- transplants, three-thirds substantiated their attitude by citing a great risk of potential complications following KTx, followed by a lack of confidence in terms of AVT efficacy in the post-kidney transplant setting. Receiving organs from aviremic donors is always preferred. However, owing to the shrinking donor pool, kidney transplant candidates may not survive until being offered one. The Polish national organization for organ transplantation (Poltransplant) report revealed that in 2021, 126 waitlisted kidney transplant candidates died without receiving a transplant; the average waitlist time for the first kidney transplant was 442 days, while highly immunized patients remained waitlisted for up to 1452 days [27]. Contrarily, there is a body of evidence to suggest that HCV NAT D+/R- transplant may not only be a favorable solution compared to remaining on HD, but it also does not necessarily entail additional complications. Importantly, HCV NAT D+/R- transplant should always be preceded by a properly informed consent process, and AVT should be administered without delay [26].

It can be further argued that the dialysis physician is not in charge of KTx matters; however, it has been proved that HD-dependent patients rely highly on their community nephrologists’ options [28]. Therefore, it is our obligation as physicians to provide patients with up-to-date and evidence-based information, enabling them to make informed decisions.

Despite the fact that Poltransplant records indicate that there was only one patient who was rejected as a donor owing to HCV-positive status, we may presume that this number may be underestimated; such potential donors may never be reported knowing the HCV serostatus [27].

Given the WHO HCV eradication target, HCV viremic donors are a finite and temporary source of additional organs. It should be used wisely for the benefit of kidney transplant candidates.

### Limitations

This study has some limitations. First, not all HD units were represented. However, the 43% response rate is similar to other surveys among HD centers, and the responding centers represent 43% of the adult HD-dependent population volume. Moreover, the consistency of the presented findings when compared to the national report data supports the idea that the study sample is representative. Our findings demonstrated practices and attitudes as they were reported, and the accuracy of actual practices and attitudes at the center could not be verified.

## 5. Conclusions

In conclusion, the present study demonstrated great disparities in HCV management practices and monitoring after virus elimination across HD centers in Poland. Differences in attitudes and HCV-care protocols may hinder the goal of HCV eradication by 2030. However, HCV eradication is no longer merely a pipe dream, and it may certainly become a reality. Nevertheless, there is a need to optimize and streamline HCV management infrastructure in patients with ESRD. A great emphasis needs to be put on a comprehensive training program dedicated to dialysis physicians to improve their poor performance in terms of fibrosis/cirrhosis evaluation and HCC surveillance in CHC patients.

## Figures and Tables

**Table 1:** Participants characteristics.

Variable		N = 112
Type of the facility		
Private	N (%)	79 (70.5)
Public	N (%)	33 (29.5)
Tertiary hospital	N (%)	13 (11.6)
Secondary hospital	N (%)	20 (17.9)
Role of the respondent within each HD center		
Head of department	N (%)	91 (81.2)
Dialysis physician	N (%)	21 (18.8)
Number of HD patients	Mean, (SD)	72.1 (32.6)
Number of anti-HCV + patients	Mean, (SD)	2.8 (2.3)
Number of patients with active viremia	Mean, (SD)	0.9 (1.43)
Specialty of the respondent		
Nephrology	N (%)	107 (95.5)
Internal medicine	N (%)	100 (89.3)
Transplant medicine	N (%)	13 (11.6)
Pulmonary disease	N (%)	4 (3.6)
Family medicine	N (%)	2 (1.8)
Diabetology	N (%)	2 (1.8)
Geriatrics	N (%)	1 (0.9)

HD; hemodialysis center.

**Table 2:** Attitudes, strategies, and obstacles in HCV care among dialysis centers in Poland.

Survey Question		Response, *n* (%)
**Do you consider patients with CHC for AVT as a routine practice?**		
Yes	N (%)	98 (91.6)
No	N (%)	9 (8.4)
**Where are subjects typically referred for AVT?**		
Infectious disease physician	N (%)	94 (83.9)
Hepatologists	N (%)	19 (17.0)
Transplant center	N (%)	2 (1.8)
**Why are hemodialysis patients with CHC not referred for AVT?**		
All patients are referred for AVT	N (%)	28 (27.7)
Patients are unwilling to undergo AVT	N (%)	41 (40.6)
Contraindication to AVT	N (%)	19 (18.8)
Short life expectancy	N (%)	11 (10.9)
Lack of awareness of AVT	N (%)	9 (8.9)
Fear of AVT-induced AEs	N (%)	7 (6.9)
Organizational matters	N (%)	6 (5.9)
Unavailability of DAAs	N (%)	6 (5.9)
Decision of the outpatient unit	N (%)	3 (3.0)
Fear of drug-drug interactions	N (%)	3 (3.0)
Low efficacy of AVT	N (%)	2 (2.0)
**Do you evaluate patients with CHC for liver fibrosis within the HD center?**		
No	N (%)	61 (60.4)
Yes	N (%)	40 (39.6)
Subject referred to AVT only	N (%)	30 (29.7)
Subject with elevated ALT only	N (%)	1 (0.9)
**Do you screen patients with CHC for HCC within the HD center?**		
No	N (%)	54 (53.5)
Yes	N (%)	47 (46.5)
**What is your HCC surveillance model for patients with CHC?**		
USG every 6 months	N (%)	24 (21.4)
AFP every 6 months	N (%)	13 (11.6)
USG every 12 months	N (%)	12 (10.7)
AFP every 12 months	N (%)	2 (1.8)
In HBV/HCV co-infection only	N (%)	6 (5.4)
In cirrhotic patients only	N (%)	5 (4.5)
If specifically recommended by the infectious disease outpatient clinic only	N (%)	2 (1.8)
**Would you offer an HCV viremic kidney to aviremic recipient?**		
Yes	N (%)	54 (48.2)
No	N (%)	54 (48.2)
Decision of the recipient	N (%)	3 (2.7)
Would require consulting	N (%)	1 (0.9)
**Where are dialyzed patients following successful HCV eradication?**		
Along with HCV-naïve patients	N (%)	49 (46.7)
On machines dedicated to hepatitis patients	N (%)	43 (40.9)
Dedicated machine following SVR	N (%)	13 (12.4)

CHC, chronic hepatitis C; HCV, hepatitis C virus; HBV, hepatitis B virus; AVT, antiviral treatment; DAAs, direct oral antiviral agents; HD, hemodialysis; USG, ultrasonography; AFP, alpha-fetoprotein; AEs, adverse events; SVR, sustained virologic response; ALT, alanine transaminase; HCC, hepatocellular carcinoma.

**Table 3:** Differences in HCV care between private and public hemodialysis centers in Poland.

Survey Question		Response, *n* (%)		
		Private	Public	
**Do you consider patients with CHC for AVT as a routine practice?**				
Yes	N (%)	71 (92.2)	27 (90.0)	*p* = 0.708
No	N (%)	6 (7.8)	3 (10.0)	
**Why are hemodialysis patients with CHC not referred for AVT?**				
All patients are referred for AVT	N (%)	23 (29.1)	5 (15.2)	χ^2^(1) = 2.42 *p* = 0.120
Patients are unwilling to undergo AVT	N (%)	29 (36.7)	12 (6.4)	χ^2^(1) = 0 *p* = 0.972
Contraindication to AVT	N (%)	12 (15.2)	7 (21.2)	χ^2^(1) = 0.60 *p* = 0.439
Short life expectancy	N (%)	8 (10.1)	3 (9.1)	*p* = 1
Lack of awareness of AVT	N (%)	3 (3.80	6 (18.2)	*p* = 0.019 *V* = 0.24
Fear of AVT-induced AEs	N (%)	5 (6.3)	2 (6.1)	*p* = 1
Organizational matters	N (%)	4 (5.1)	2 (6.1)	*p* = 1
Unavailability of DAAs	N (%)	4 (5.1)	2 (6.1)	*p* = 1
Decision of the outpatient unit	N (%)	1 (1.3)	2 (6.1)	*p* = 0.207
Fear of drug-drug interactions	N (%)	2 (2.5)	1 (3.0)	*p* = 1
Low efficacy of AVT	N (%)	2 (2.5)	0 (0.0)	*p* = 1
**Do you evaluate patients with CHC for liver fibrosis within the HD center?**				
No	N (%)	44 (62.0)	17 (56.7)	χ^2^(1) = 0.20 *p* = 0.656
Yes	N (%)	27 (38.0)	13 (43.3)	
Only referred for antiviral treatment	N (%)	23 (32.4)	7 (23.3)	
Only patients with abnormal aminotransferases	N (%)	0 (0.0)	1 (3.3)	
**Do you screen patients with CHC for HCC within the HD center?**				
Yes	N (%)	30 (42.9)	17 (54.8)	χ^2^(1) = 1.24 *p* = 0.266
No	N (%)	40 (57.1)	14 (45.2)	
**What is your HCC surveillance model for patients with CHC?**				
Ultrasound every 12 months	N (%)	8 (10.1)	4 (12.1)	*p* = 0.746
Ultrasound every 6 months	N (%)	13 (16.5)	11 (33.3)	χ^2^(1) = 3.94 *p* = 0.047 *V* = 0.19
AFP every 12 months	N (%)	1 (1.3)	1 (3.0)	*p* = 0.504
AFP every 6 months	N (%)	5 (6.3)	8 (24.2)	*p* = 0.019 *V* = 0.26
Patients with HBV co-infection only	N (%)	6 (7.6)	0 (0.0)	*p* = 0.177
Patients with cirrhosis only	N (%)	3 (3.8)	2 (6.1)	*p* = 0.630
If specifically recommended by the infectious disease outpatient clinic only	N (%)	1 (1.3)	1 (3.0)	*p* = 0.504
**Where are dialyzed patients following successful HCV eradication?**				
Along with HCV naïve patients	N (%)	31 (42.5)	18 (56.2)	χ^2^ (2) = 1.96 *p* = 0.352
On machines dedicated to hepatitis patients	N (%)	33 (45.2)	10 (31.3)	
Dedicated machine following SVR	N (%)	9 (12.3)	4 (12.5)	

CHC, chronic hepatitis C; AVT, antiviral treatment; DAAs, direct oral antiviral agents; HD, hemodialysis; USG, ultrasonography; AFP, alpha-fetoprotein; HCV, hepatitis C virus; AEs, adverse events; HCC, hepatocellular carcinoma.

**Table 4:** Differences in HCV care between tertiary and secondary hemodialysis centers in Poland.

Survey Question		Response, *n* (%)		
		Tertiary	Secondary	
**Do you consider patients with CHC for AVT as a routine practice?**				
Yes	N (%)	11 (100.0)	16 (84.2)	*p* = 0.279
No	N (%)	0 (0.0)	3 (15.8)	
**Why are hemodialysis patients with CHC not referred for AVT?**				
All patients are referred for AVT	N (%)	3 (23.1)	2 (10.0)	*p* = 0.360
Patients are unwilling to undergo AVT	N (%)	4 (30.8)	8 (40.0)	*p* = 0.719
Contraindication to AVT	N (%)	2 (15.4)	5 (25.0)	*p* = 0.676
Short life expectancy	N (%)	1 (7.7)	2 (10.0)	*p* = 1
Lack of awareness of AVT	N (%)	2 (15.4)	4 (20.0)	*p* = 1
Fear of AVT-induced AEs	N (%)	0 (0.0)	2 (10.0)	*p* = 1
Organizational matters	N (%)	0 (0.0)	2 (10.0)	*p* = 0.508
DAAs unavailability	N (%)	0 (0.0)	2 (10.0)	*p* = 0.508
Decision of the outpatient unit	N (%)	1 (7.7)	1 (5.0)	*p* = 1
Fear of drug-drug interactions	N (%)	0 (0.0)	1 (5.0)	*p* = 1
Low efficacy of AVT	N (%)	0 (0.0)	0 (0.0)	-
**Do you evaluate patients with CHC for liver fibrosis within the HD center?**				
No	N (%)	1 (9.1)	16 (84.2)	*p* < 0.001 *V* = 0.74
Yes	N (%)	10 (90.9)	3 (15.8)	
Only referred for antiviral treatment	N (%)	5 (45.5)	2 (10.5)	
Only patients with abnormal aminotransferases	N (%)	1 (9.1)	0 (0.0)	
**Do you screen patients with CHC for HCC within the HD center?**				
Yes	N (%)	7 (63.6)	10 (50.0)	χ^2^(1) = 1.11 *p* = 0.293
No	N (%)	4 (36.4)	10 (50.0)	
**What is your HCC surveillance model for patients with CHC?**				
Ultrasound every 12 months	N (%)	4 (30.8)	0 (0.0)	*p* = 0.017 *V* = 0.46
Ultrasound every 6 months	N (%)	4 (30.8)	7 (35.0)	*p* = 1
AFP every 12 months	N (%)	1 (7.7)	0 (0.0)	*p* = 0.394
AFP every 6 months	N (%)	5 (38.5)	3 (15.0)	*p* = 0.213
Patients with HBV co-infection only	N (%)	0 (0.0)	0 (0.0)	-
Patients with cirrhosis only	N (%)	0 (0.0)	2 (10.0)	*p* = 0.508
If specifically recommended by the infectious disease outpatient clinic only	N (%)	1 (7.7)	0 (0.0)	*p* = 0.394
**Where are dialyzed patients following successful HCV eradication?**				
Along with HCV naïve patients	N (%)	6 (50.0)	12 (66.6)	*p* = 0.882
On machines dedicated to hepatitis patients	N (%)	4 (33.3)	6 (33.3)	
Dedicated machine following SVR	N (%)	2 (16.7)	2 (11.1)	

CHC, chronic hepatitis C; AVT, antiviral treatment; DAAs, direct oral antiviral agents; HD, hemodialysis; AFP, alpha-fetoprotein; HCV, hepatitis C virus; AEs, adverse events; HCC, hepatocellular carcinoma.

**Table 5:** Solutions on local and national levels to improve HCV management in hemodialysis centers in Poland.

Local Level	National Level
Refrain from isolating HCV-positive subjects and those who achieved SVR in the hemodialysis setting	Dialysis physicians taking over the AVT in patients on HD and with CHC.
Comprehensive hepatitis training among dialysis physicians with the main focus being put on the following: - current treatment options and simplified approach towards AVT, - benefits of AVT, - contraindication for AVT, - tools for fibrosis evaluation and predictive cut-off values, - need for HCC surveillance following SVR in subjects with advanced fibrosis/cirrhosis	DAAs to be available in HD centers for CHC management. National consultants could be engaged to issue a recommendation on obligatory fibrosis evaluation in all patients on hemodialysis and with CHC, followed by HCC surveillance. Possibility of consulting with hepatitis expert needs to be assured (remotely, if possible). Amount of paperwork needs to be reduced in order to facilitate DAAs administration in HD centers.
Training on infectious control strategies among dialysis staff with regular audits to assure compliance.	
Regular screening for possible HCV reinfection and/or outbreaks within HD centers	
The root cause of patients’ reluctance to AVT needs to be further investigated and addressed with respective measures depending on the outcome, e.g., educating patients by the trained dialysis nurse.	

CHC, chronic hepatitis C; AVT, antiviral treatment; DAAs, direct oral antiviral agents; HD, hemodialysis; AFP, alpha-fetoprotein; HCV, hepatitis C virus; HCC, hepatocellular carcinoma, SVR; sustained virologic response.

## Data Availability

Data supporting the results reported in the article can be found under the following https://doi.org/10.17632/ch8rh343m9.1.
